# Basket cell contributions to the generation of theta rhythms in model hippocampal CA1 networks

**DOI:** 10.1186/1471-2202-12-S1-P305

**Published:** 2011-07-18

**Authors:** Katie A Ferguson, Carey YL Huh, Bénédicte Amilhon, Sylvain Williams, Frances K Skinner

**Affiliations:** 1 Physiology, University of Toronto, Toronto, Ontario, M5S 1A1, Canada; 2 Toronto Western Research Institute, University Health Network, Toronto, Ontario, M5T 2S8, Canada; 3 Medicine (Neurology), Physiology, Institute of Biomaterials and Biomedical Engineering, University of Toronto, Toronto, Ontario, M5S 1A1, Canada; 4 Psychiatry, Douglas Mental Health University Institute, McGill University, Montreal, Quebec, H4G 1X6, Canada

## 

Theta oscillations are one of the most prominent and well-studied clocking mechanisms detected in the mammalian brain. Recorded from the hippocampus during R.E.M. sleep and exploratory behavior, these 3-12 Hz rhythms are thought to play a lead role in spatial navigation, episodic memory, and the timing of place cell firing [1]. Although these oscillations have been heavily studied, the mechanism(s) responsible for the generation of these rhythms remains unknown. A popular theory hypothesizes that pacemaker neurons in the medial septum drive hippocampal rhythms [1] , but recent research shows that in an intact hippocampus preparation *in vitro*, the CA1 hippocampal region possesses the necessary circuitry to generate intrinsic theta rhythms [2]. To determine the mechanism(s) underlying the generation of these CA1 hippocampal theta rhythms, we created a mathematical network model.

Our mathematical network model is composed of four types of cells: pyramidal cells, fast-spiking parvalbumin-positive basket cells (PV+BCs), slow-spiking cholecystokinin-positive basket cells (CCK+BCs), and oriens – lacunosum-moleculare (O-LM) interneurons. Each cell type is represented by a single-compartment, conductance-based model, and intrahippocampal connections among the chosen cell types are modeled based on experimental data of known connectivities. In addition, intracellular data recorded from the CA1 region of the intact hippocampus *in vitro* was used in combination with mathematical extraction techniques [3,4] to determine the balance of synaptic excitation and inhibition in individual cell types during the theta rhythm. Thus, experimental recordings, data analysis, and modeling were combined to generate an understanding of the mechanism(s) involved in the CA1 hippocampal theta rhythm.

Our network model produces robust theta rhythms and cellular phase relationships in accordance with the experimental data. Interestingly, we find that inhibitory input imposed on pyramidal cells from the PV+BCs is a critical component in the production of these theta rhythms because the existence of the rhythm is most sensitive to these inhibitory conductances. This finding is surprising, as research has focused on the role of PV+BCs in faster gamma rhythms (20-100 Hz). In addition, we extracted mean synaptic conductance values from intracellular recordings of PV+BC and somatostatin-expressing (putative O-LM) interneuron activity. These synaptic values indicate that from the quiescent relative to the active state of the CA1 theta rhythm, the PV+BCs undergo a more significant reduction in inhibition than the putative O-LM interneurons. Optogenetics will be used to test predictions about the role of individual interneuron types in the generation of CA1 hippocampal theta rhythms.
